# Human predecidual stromal cells are mesenchymal stromal/stem cells and have a therapeutic effect in an immune-based mouse model of recurrent spontaneous abortion

**DOI:** 10.1186/s13287-019-1284-z

**Published:** 2019-06-14

**Authors:** Raquel Muñoz-Fernández, Claudia De La Mata, Francisco Requena, Francisco Martín, Pablo Fernandez-Rubio, Tatiana Llorca, Maria José Ruiz-Magaña, Carmen Ruiz-Ruiz, Enrique G. Olivares

**Affiliations:** 1 Instituto de Biopatología y Medicina Regenerativa, Centro de Investigación Biomédica, Universidad de Granada, Granada, Spain; 2 Departamento de Estadística e Investigación Operativa, Universidad de Granada, Granada, Spain; 3 Human DNA Variability Department, GENYO - Centre for Genomic and Oncological Research (Pfizer/University of Granada/Andalusian Regional Government), PTS Granada, Granada, Spain; 4 Departamento de Bioquímica y Biología Molecular III e Inmunología, Facultad de Medicina, Universidad de Granada, Avenida de la Investigación, 11, 18016 Granada, Spain; 5 Unidad de Gestión Clínica Laboratorios, Hospital Universitario San Cecilio, Granada, Spain

**Keywords:** Clone cells, Decidua, Immune tolerance, Immunotherapy, Mesenchymal stromal cells, Predecidual stromal cells, Recurrent abortion

## Abstract

**Background:**

Human decidual stromal cells (DSCs) are involved in the maintenance and development of pregnancy, in which they play a key role in the induction of immunological maternal–fetal tolerance. Precursors of DSCs (preDSCs) are located around the vessels, and based on their antigen phenotype, previous studies suggested a relationship between preDSCs and mesenchymal stromal/stem cells (MSCs). This work aimed to further elucidate the MSC characteristics of preDSCs.

**Methods:**

We established 15 human preDSC lines and 3 preDSC clones. Physiological differentiation (decidualization) of these cell lines and clones was carried out by in vitro culture with progesterone (P4) and cAMP. Decidualization was confirmed by the change in cellular morphology and prolactin (PRL) secretion, which was determined by enzyme immunoassay of the culture supernatants. We also studied MSC characteristics: (1) In mesenchymal differentiation, under appropriate culture conditions, these preDSC lines and clones differentiated into adipocytes, osteoblasts, and chondrocytes, and differentiation was confirmed by cytochemical assays and RT-PCR. (2) The expression of stem cell markers was determined by RT-PCR. (3) Cloning efficiency was evaluated by limited dilution. (4) Immunoregulatory activity in vivo was estimated in DBA/2-mated CBA/J female mice, a murine model of immune-based recurrent abortion. (5) Survival of preDSC in immunocompetent mice was analyzed by RT-PCR and flow cytometry.

**Results:**

Under the effect of P4 and cAMP, the preDSC lines and clones decidualized in vitro: the cells became rounder and secreted PRL, a marker of physiological decidualization. PreDSC lines and clones also exhibited MSC characteristics. They differentiated into adipocytes, osteoblasts, and chondrocytes, and preDSC lines expressed stem cell markers OCT-4, NANOG, and ABCG2; exhibited a cloning efficiency of 4 to 15%; significantly reduced the embryo resorption rate (*P* < 0.001) in the mouse model of abortion; and survived for prolonged periods in immunocompetent mice. The fact that 3 preDSC clones underwent both decidualization and mesenchymal differentiation shows that the same type of cell exhibited both DSC and MSC characteristics.

**Conclusions:**

Together, our results confirm that preDSCs are decidual MSCs and suggest that these cells are involved in the mechanisms of maternal–fetal immune tolerance.

**Electronic supplementary material:**

The online version of this article (10.1186/s13287-019-1284-z) contains supplementary material, which is available to authorized users.

## Background

Decidual stromal cells (DSCs), which constitute the main cellular component of human decidua, show activities that appear to play an important role in the embryo implantation [1], development of pregnancy [2], and maternal–fetal immune tolerance [3–7]. These cells originate from perivascular fibroblastic precursors, which are detected in the gestational (decidua) as well as the nongestational endometrium [8, 9]. Under the effect of ovarian hormones, these DSC precursors leave the vessels and differentiate into decidualized cells, which then spread throughout the stroma in the late luteal phase (decidualization). Decidualized cells change from a fibroblastic to a rounder shape and secrete prolactin (PRL) [10–12]. These differentiated cells are discarded during menstruation; however, if pregnancy takes place, the decidualization process continues because of the effect of pregnancy hormones [8, 9]. Olivares et al. [13] introduced the term “predecidual stromal cells” (preDSCs) to designate the precursors of DSCs, and this term was later used by other authors [10, 14–16]. PreDSCs can be isolated from both the endometrium and decidua and cultured in vitro. Moreover, they decidualize, as in vivo, when incubated with progesterone (P4) and cAMP, changing to a rounder cell shape and secreting factors which are considered distinctive markers of decidualization, including PRL [13, 17].

The isolation and maintenance of highly purified human preDSC lines in culture allowed us to analyze the antigen phenotype of these cells and study their functions [3–5, 18, 19]. Based on the perivascular location of preDSCs, together with their expression of pericyte markers and angiogenic factors, their phagocytic activity, and their capacity to contract in vitro, we demonstrated a close relationship between preDSCs and pericytes [19], contractile cells that surround microvascular endothelial cells [20, 21]. Furthermore, experimental evidence showed strong similarities between pericytes and mesenchymal stromal/stem cells (MSCs), and some have proposed that they constitute the same cell population [22–24]. In this connection, we previously confirmed that the antigen phenotype of preDSCs is compatible with MSCs [5, 19, 25]. Functions such as cell contractility, resistance to apoptosis, and inhibition of lymphocyte apoptosis are also shared by MSCs and preDSCs [18, 26–30]. Further evidence of a close relationship between preDSCs and MSCs was also reported by Dimitrov et al. [31].

Mesenchymal stromal/stem cells exhibit immunoregulatory activities in vivo and in vitro that show promise in the treatment of inflammatory and autoimmune diseases [32]. Therefore, to further elucidate the relationship between preDSCs and MSCs, the present study was designed to investigate the in vivo effects of preDSC lines in a murine model of immune system-mediated human recurrent spontaneous abortion. This is a well-known model in which CBA female mice are mated to DBA2 male mice, and pregnant CBA/2 animals have a high incidence of embryo resorption. Different immune cells and immunological mechanisms are involved in this resorption [33, 34]. We considered this murine model appropriate to investigate both the potential regulatory effect of preDSCs in immune-based human recurrent abortion and the role of preDSCs in maintaining maternal–fetal immune tolerance.

## Materials and methods

### Samples

For the preDSC lines, samples from elective vaginal terminations of first-trimester pregnancies (6–11 weeks) were collected from 15 healthy women aged 20–30 years. We excluded women who were using any medication or with infectious, autoimmune, or other systemic or local disease. None of the abortions was pharmacologically induced. The specimens were obtained by suction curettage at the Clínica El Sur in Málaga or the Clínica Ginegranada in Granada. For the 3 human foreskin fibroblast (HFF) lines, foreskin samples were obtained from patients at the Hospital Universitario San Cecilio in Granada.

### Isolation and culture of preDSC and HFF lines

We used the preDSC lines obtained in recent work as reported earlier [19]. These lines consisted of a highly purified, uniform, adherent cell population in which almost all cells expressed the endometrial stromal cell marker CD10 [35], together with CD13, CD29, CD44, CD73, CD90, CD105, α-smooth muscle (SM) actin, nestin, podoplanin, and vimentin, and lacked CD15, CD19, CD34, CD45, CD62P, and HLA-DR expression. This antigen phenotype is fully compatible with that of bone marrow MSCs [19, 36] (Table 1). To establish HFF lines from the human foreskin, we used the method described by Kimatrai and colleagues [28] to establish preDSC lines from the decidua. Briefly, foreskin samples were minced between two scalpels in a small volume of PBS. The suspension was mixed with a solution of 5 mg/mL collagenase V (Sigma-Aldrich, St. Louis, MO, USA) for 30 min at 37 °C. The suspension was diluted in PBS and centrifuged at 425*g* for 10 min. The cell pellet was suspended in PBS and centrifuged on Ficoll-Paque (Sigma-Aldrich) for 20 min at 600*g*. Foreskin cells were collected from the interface, suspended in PBS, and washed. The resulting suspension was incubated in culture flasks for 24 h at 37 °C with 5% CO_2_ in Opti-MEM (minimum essential medium) (Invitrogen, Grand Island, NY, USA) supplemented with 3% fetal calf serum (FCS) (Invitrogen), 100 IU/mL penicillin, 100 μg/mL streptomycin, and 0.25 μg/mL amphotericin (Sigma-Aldrich). After overnight incubation to allow adherent cells to attach to the flask, nonadherent cells in the supernatant were discarded. The medium was then replaced and changed thereafter twice a week. After 1–3 weeks, adherent cells were morphologically uniform and covered the whole surface of the 25-cm^2^ culture flask. Cells were split with a trypsin-EDTA solution of 0.25% (Sigma-Aldrich) when they were 90 to 100% confluent. Although the different cell lines are referred to generically as preDSCs or HFFs, in experiments with several lines of the same type of cell, we used a specific designation for each line (e.g., preDSC1, preDSC2). For this study, 15 preDSC and 3 HFF lines were obtained (each from a different sample) and were always used between 3 and 8 weeks after collection (up to 5 passages). The maternal origin of each preDSC line was confirmed by comparison with its corresponding trophoblast obtained from the same sample, using short tandem repeat markers and quantitative-fluorescent PCR (Devyser AB, Hägersten, Sweden).

**Table 1:** Antigen expression by preDSC and bone marrow MSC lines obtained in the same conditions [19, 36]

Antigen	Flow cytometry reactions	
	preDSCs	MSCs
CD10^a^	**+**	**+**
CD13^a^	**+**	**+**
CD15	–	–
CD19^b^	–	–
CD29^a^	**+**	**+**
CD31	–	–
CD34^b^	–	–
CD44^a^	**+**	**+**
CD45^b^	–	–
CD54	+	+
CD62P	–	–
CD73^a,b^	**+**	**+**
CD90^a,b^	**+**	**+**
CD105^a,b^	**+**	**+**
CD106	+/−^c^	+/−^c^
CD140b	+	ND
CD146	+	+
CD271	+	+
AP	+	ND
α-SM actin^a^	**+**	**+**
HLA-G	+	+/−
HLA-DR^b^	–	–
MFGE8	+	ND
Nestin^a^	**+**	**+**
OCT3/4	+	+
Podoplanin^a^	**+**	**+**
STRO-1	+	+
Vimentin^a^	**+**	ND
W5C5	+	+

*ND* not done

^a^Antigens expressed by more than 95% of cells

^b^Antigens meeting the minimal criteria for identification as MSC [37]

^c^Some lines expressed the antigen and some did not

### Cell cloning

We used three preDSC clones obtained in earlier work [19]. Predecidual stromal cell clones were obtained from preDSC lines by limiting dilution in 96-well plates, using complete Opti-MEM supplemented with 10% FCS. Three days after cell seeding, the plates were checked and wells with only one cell were selected. After 2 weeks, single cells had formed colonies which we then trypsinized and seeded into 24-well plates for culture in complete Opti-MEM supplemented with 3% FCS.

### Decidualization

To induce decidualization, preDSC lines or clones were treated with 300 nM P4 and 500 μM dibutyryl cAMP (Sigma-Aldrich) for 15 days. Decidualization was verified by PRL secretion and changes in cell morphology from a fibroblastic to a round shape, as observed with light microscopy. The presence of PRL was verified with an electrochemiluminescence immunoassay (Roche, Indianapolis, IN, USA). The assays were performed according to the manufacturer’s instructions, and all samples were tested in duplicate.

### Mesenchymal differentiation

For osteogenic differentiation, preDSCs were plated on a fresh tissue culture dish at 80 to 90% confluency in Opti-MEM. The cells were allowed to attach for a minimum of 24 h and rinsed with PBS. Complete osteogenic differentiation medium (Opti-MEM supplemented with 10 nM ascorbic acid, 10 nM β-glycerol phosphate, 100 nM dexamethasone [Sigma-Aldrich], and 3% FCS) was added, and the cells were incubated at 37 °C with 5% CO_2_ with humidity. Every 3 days, the medium was replaced with fresh complete osteogenic differentiation medium. Osteogenesis required approximately 21–28 days and was verified by the formation of tightly packed, elongated osteoblasts. In addition, the mineralized matrix was detected by alizarin staining (Sigma-Aldrich). For adipogenic differentiation, cells were plated on 5% FCS-Opti-MEM in culture dishes and incubated at 37 °C in 5% CO_2._ Two days after confluency, the cells were stimulated with induction medium consisting of 100 μM 3-isobutyl-1-methylxanthine, 1 μM dexamethasone, 100 μM indomethacin (Sigma-Aldrich), and 10 μg/mL insulin (Novo Nordisk, Bagsvaerd, Denmark). Full differentiation was usually achieved by day 15. Lipid droplets were visualized with oil red O solution (Sigma-Aldrich). For chondrogenic differentiation, adherent cell colonies were trypsinized and counted, and the medium was replaced with serum-free Opti-MEM supplemented with 10 ng/mL TGF-β1 (Sigma-Aldrich). Aliquots of 2 × 10^5^ cells in 1 mL medium were then centrifuged in conical polypropylene tubes. The pelleted cells were incubated at 37 °C in 5% CO_2_. Within 24 h after incubation, the cells formed an aggregate that did not adhere to the walls of the tube. The medium was changed every 2 or 3 days, and the cell aggregates were obtained at intervals of up to 21 days. To detect proteoglycans, 4-μm-thick frozen sections of aggregates were stained with safranin O solution (Sigma-Aldrich).

### Colony-forming unit test

Briefly, preDSCs were plated (100 cells/cm^2^) in 6-well plates with 2 mL Opti-MEM supplemented with 3% FCS, 100 IU/mL penicillin, 100 μg/mL streptomycin, and 0.25 μg/mL amphotericin. The plates were incubated at 37 °C with 5% CO_2_ for 15 days. Colonies were monitored daily with light microscopy to verify they were derived from single cells. The colonies were stained with 0.1% crystal violet (Sigma-Aldrich) for 5 min at room temperature and washed with distilled water. Only colonies with more than 50 cells were counted. The percent cloning efficiency (CE) was calculated with the following formula: CE (%) = number of colonies/number of cells seeded.

### Reverse transcription polymerase chain reaction

The primers used in this study, all from the Instituto de Parasitología y Biomedicina, Granada, Spain, are detailed in Table 2. Octamer-binding transcription factor 4 (OCT-4) primers were obtained from Integrated DNA Technologies (Leuven, Belgium). To distinguish bona fide OCT-4 transcripts from pseudogene transcripts, we performed RT-PCR assays with two different forward primers and one intron-spanning reverse primer (Oct4R). One of the forward primers (Oct4FP) recognizes an exclusive polymorphism of OCT-4, which makes it possible to distinguish its corresponding transcript from pseudogene transcripts. The other forward primer (Oct4F) recognizes a nonhomologous region of pseudogenes [38].

**Table 2:** Primer sequences used for RT-PCR

mRNA	Oligonucleotide primers	PCR product size (bp)
ATP-binding cassette subfamily G member 2 (ABCG2)	5′-CCCATCCTGACCTCCAGCCG-3′ (F) 5′-TTGAGTGGGCACAGCACGCA-3′ (R)	127
Alkaline phosphatase (AP)	5′-TGGAGCTTCAGAAGCTCAACACCA-3′ (F) 5′-ATCTCGTTGTCTGAGTACCAGTCC-3′ (R)	338
β2-microglobulin	5′-CTCGCGCTACTCTCTCTCTTTCTGG-3′ (F) 5′-TCTACATGTCTCGATCCCACTTAA-3′ (R)	335
Collagen II	5′-TTTCCCAGGTCAAGATGGTC-3′ (F) 5′-CTTCAGCACCTGTCTCACCA-3′ (R)	159
Human cytochrome B (Cyt B)	5′-CCCATACATTGGGACAGACC-3′ (F) 5′-GACGGATCGGAGAATTGTGT-3′ (R)	394
Murine Cyt B	5′-TCGCGGCCCTAGCAATCGTT-3′ (F) 5′-TGGTTGGCCCCCAATTCAGGT-3′(R)	461
NANOG	5′-TGCTGGACTGAGCTGGTTGCC-3′ (F) 5′-AGCAAGGCAAGCTTTGGGGACA-3′ (R)	221
Octamer-binding transcription factor 4 (OCT-4)	Oct4F: 5′-AGCCCTCATTTCACCAGGCC-3′(F) Oct4FP: 5′-GATGGCGTACTGTGGGCCC-3′ (F) Oct4R: 5′-TGGGACTCCTCCGGGTTTTG-3′ (R)	456 195
Osteopontin	5′-CTAGGCATCACCTGTGCCATACC-3′ (F) 5′-CAGTGACCAGTTCATCAGATTCATC-3′ (R)	373
Peroxisome proliferator-activated receptor γ2 (PPARγ2)	5′-GCTGTTATGGGTGAAACTCTG-3′ (F) 5′-ATAAGGTGGAGATGCAGGCTC-3′ (R)	351

*F* forward, *R* reverse

Total RNA was extracted from cells with the TRIzol isolation method, and cDNA was synthesized with Oligo-dT primers and Moloney murine leukemia virus H minus ribonuclease reverse transcriptase (Invitrogen) according to the manufacturer’s protocol. For RT-PCR, we used a 2720 Thermal Cycler (Applied Biosystems, Darmstadt, Germany). The reaction mixture (total volume 20 μL) contained cDNA (equivalent of 100 ng RNA), 200 nM deoxy-NTPs (Biotools, Madrid, Spain), 800 nm of each primer, and 0.5 U GoTaq polymerase (Biotools). After incubation for 3 min at 95 °C, each cycle consisted of 95 °C for 30 s, 55 °C for 45 s, and 72 °C for 45 s, for a total of 35 cycles. The RT-PCR products were size-separated on ethidium bromide-stained 2% agarose gels (PanReact AppliChem, Barcelona, Spain), and a 100-bp DNA ladder was included in each run.

### Plasmids, lentiviral constructs, vector production, titration, and preDSC transduction

The HIV packaging (pCMVΔR8.91) and the vesicular stomatitis virus G glycoprotein (VSVg) (pMD.G) plasmids were kindly provided by D. Trono (University of Geneva, Geneva, Switzerland). The packaging plasmid pCMVΔR8.91 encodes *gag*, *pol*, *tat*, and *rev* genes. The pMD.G plasmid encodes *VSVg*. The CEWP lentiviral vectors express an enhanced green fluorescent protein (GFP) through the cytomegalovirus (CMV) promoter [39]. Lentiviral vectors were produced by cotransfection of 293T kidney cells (human embryonic kidney cells, CRL-11268, ATCC) as previously described [40]. Briefly, the three plasmids encoding the vector genome (CEWP), the gag-pol-tat-rev proteins (packaging plasmid- pCMVΔR8.91), and the envelope glycoprotein (pMD.G) were transfected into 293T cells with Lipofectamine 2000 (Invitrogen). Viral supernatants were collected and filtered through a 0.45-μm filter. Vector particles were concentrated by ultrafiltration at 2000×*g*, 4 °C with 100-kDa centrifuge filter devices as previously described [41] (Amicon Ultra-15, Millipore, Billerica, MA, USA). Vector supernatants were immediately frozen at − 80 °C or used to transduce preDSCs. Briefly, preDCSs were detached from the culture flask, washed with PBS, resuspended in vector supernatant at a multiplicity of infection of 10, and left in the incubator for 5–6 h. The viral supernatant was removed and replaced with fresh Opti-MEM culture medium. Four to 6 days later, the CEWP-transduced preDSCs were analyzed by FACS for GFP expression. The antigen phenotype, cell viability, and proliferation of transduced preDSCs were equivalent to those of nontransduced preDSCs.

### Mice

Eight- to 10-week-old BALB/c, DBA/2, and CBA/J mice (Janvier Labs, Le Genest-Saint-Isle, France) were housed in our center’s animal facility (Centro de Investigación Biomédica, Universidad de Granada) in stable humidity and temperature conditions on a 12:12-h light/dark cycle, with free access to food and water. All necessary steps were taken to ensure maximum comfort of the animals and compliance with current regulations on the maintenance and use of experimental animals.

### Murine model of recurrent abortion

Virgin female CBA mice were mated to male BALB/c mice (control mating combination) or DBA2 male mice (abortion-prone mating combination). For each combination, we established three groups: control PBS (intraperitoneal injection of 0.5 mL PBS), control HFFs (intraperitoneal injection of 10^6^ HFFs in 0.5 mL PBS), and preDSC (intraperitoneal injection of 10^6^ preDSCs in 0.5 mL PBS). HFFs were used in these experiments as a negative control for preDSCs, because both types of cell are fibroblastic cells. Pregnancy was verified by the presence of a vaginal plug (gestation day 0.5), and 1.5 days later, we injected PBS or preDSCs. The animals were killed on day 14 of gestation, and the number of embryo implants and embryo resorptions was recorded in each pregnant mouse. The results were expressed as resorption rate: the percentage of reabsorbed embryos (no embryo in the implantation site) referred to the total number of implanted embryos.

### Detection of human preDSCs in the mice tissues

The presence of preDSCs after intraperitoneal injection in pregnant CBA/J mice in the murine model of recurrent abortion was determined by RT-PCR with human B cytochrome (Cyt B) primers for human mitochondrial DNA. Pellets (0.5 mL) of centrifuged blood or small pieces of dispersed spleen tissue were suspended in 0.5 mL of a solution of 0.1 M EDTA and 50 Mm TRIS (pH = 8) containing 0.5% SDS and 1 mg/mL proteinase K (Roche, Roche Diagnostics, Basel, Switzerland) and incubated overnight at 55 °C. Fifty microliters of a 3-M sodium acetate solution (pH 7) and then 0.5 mL of a phenol-chloroform (1:1) solution were added. The samples were vortexed and centrifuged at 16,200*g* for 5 min at room temperature. Four hundred microliters of the upper aqueous phase was collected and mixed with 350 μL isopropanol, and the mixture was centrifuged at 16,200*g* for 5 min at room temperature. The supernatant was discarded, and the pellet was washed with absolute ethanol at 16,200*g* for 5 min at room temperature. The supernatant was again discarded, and the pellet was suspended in milliQ water and incubated overnight at 55 °C. Polymerase chain reaction was done with a 2720 thermal cycler (Applied Biosystems). The PCR mixture contained 800 ng DNA, 200 μM dNTPs, and 10 μM of each primer in a total volume of 50 μL. The PCR reactions were run with an initial step at 95 °C for 10 min followed by 35 cycles at 94 °C for 1 min, 60 °C for 1 min, and 72 °C for 1 min.

We also injected 10^6^ CEWP-transduced preDSCs suspended in PBS intraperitoneally in virgin female BALB/c mice. The animals were killed on different days, and the blood, spleen, and inguinal lymph nodes were collected. Blood was diluted in PBS and centrifuged on Ficoll-Paque (Sigma-Aldrich). Lymph node and spleen tissues were disaggregated with 5 mg/mL collagenase V (Sigma-Aldrich) for 30 min at 37 °C, washed, suspended in PBS, and centrifuged on Ficoll-Paque (Sigma-Aldrich). Cells were collected from the interface, suspended in PBS, and analyzed in a FACScan cytometer (BD Biosciences, San Diego, CA, USA). Tissues from BALB/c mice in which nontransduced preDSCs had been injected were used as the negative control for flow cytometry.

### Statistical analysis

The figures illustrate the results for a single experiment representative of three or more separate assays. All experiments were done in triplicate or quadruplicate. The Wilcoxon test was used to compare the results for the changes in morphology and PRL secretion. To study recurrent abortion, the results were analyzed with the chi-squared test corrected for a cluster sampling design (Rao–Scott correction) [42], as uncorrected chi-squared or Fisher’s exact tests, used by other authors, may yield misleading results. Values of *P* < 0.05 were considered significant.

## Results

### MSC characteristics of preDSC lines

**Fig. 1:**
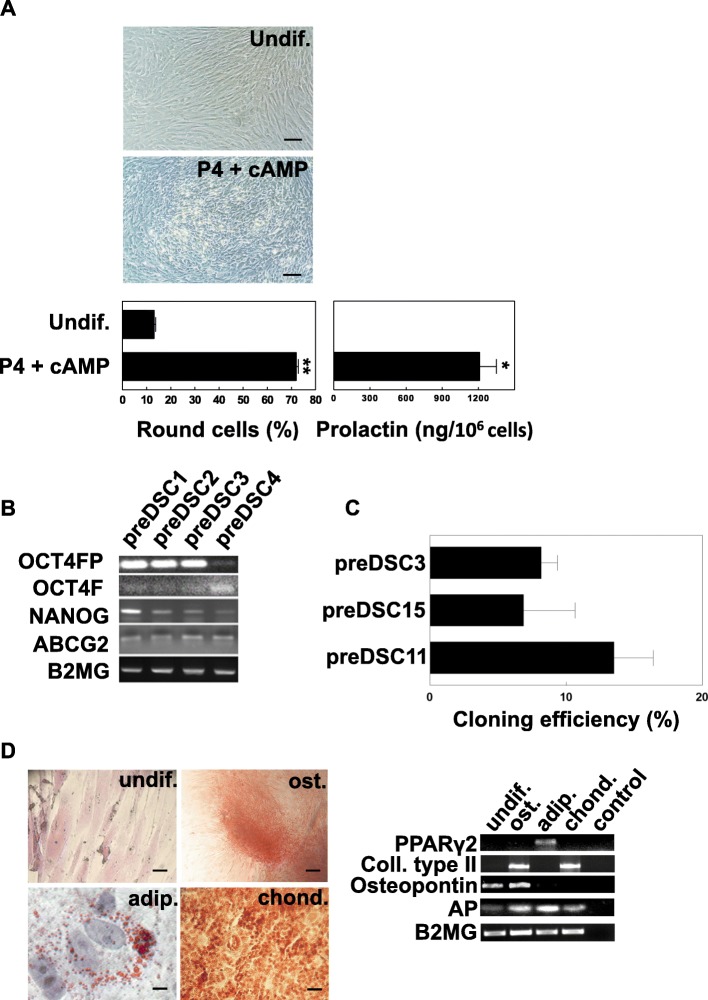
Characteristics of preDSC lines. **a** Decidualization. After 14 days of culture with P4 and cAMP, undifferentiated preDSCs (Undif.) changed from a fibroblastic to a rounder shape (left) and secreted PRL (right). The bars show the percentage of round cells and the secretion of PRL by a preDSC line (mean ± SD of three independent determinations). Scale bar, 50 μm. **b** Detection by RT-PCR of pluripotent stem cell markers in four preDSC lines. **c** Cloning efficiencies of the three preDSC lines. **d** Mesenchymal differentiation of a preDSC line. Undifferentiated preDSCs (undif.), osteogenic (ost.), adipogenic (adip.), and chondrogenic (chond.) differentiated preDSCs. Differentiation was documented with alizarin staining (ost.), oil red O solution (adip.), and safranin O (chond.). Scale bar, 50 μm. Molecular markers of differentiation were detected by RT-PCR: PPARγ2 (adip.), collagen type II (chond.), osteopontin, and AP (ost.). AP and osteopontin were also positive in undifferentiated preDSC lines [25, 28]. Beta-2 microglobulin (B2MG) was used as the loading control. The bars represent mean ± SD of three independent determinations. These experiments were done in 4 independent replications. *P < 0.001, **P < 0.0005

The preDSC lines were composed of a highly purified uniform population of proliferating fibroblast-shaped adherent cells [19]. These cells exhibited an antigen phenotype compatible with that of MSCs [19, 36] (Table 1). In cultures with cAMP and P4, preDSC lines decidualized, changing their morphology from a fibroblastic to a rounder shape and secreting PRL (Fig. 1a). Furthermore, by RT-PCR, we detected the pluripotent stem cell markers OCT-4, NANOG, and ABCG2 in preDSCs (Fig. 1b)—markers also associated with MSCs [43]. Detecting OCT-4 transcripts by RT-PCR is always a challenge, since artifacts are easily induced by the amplification of many OCT-4-like transcripts originating from the expression of processed and nonprocessed pseudogenes. Both forward primers, i.e., OCT-4-specific OCT-4FP and OCT-4F, which also amplifies pseudogenes, were positive in all 4 preDSC lines. The colony-forming efficiency of the preDSC lines ranged between 4 and 15% (Fig. 1c). Under appropriate culture conditions, preDSCs differentiated into the three different mesenchymal lineages—adipocytes, osteoblasts, and chondrocytes—as confirmed by cytochemical assays and RT-PCR (Fig. 1d).

### PreDSC clones exhibited both DSC and MSC characteristics

**Fig. 2:**
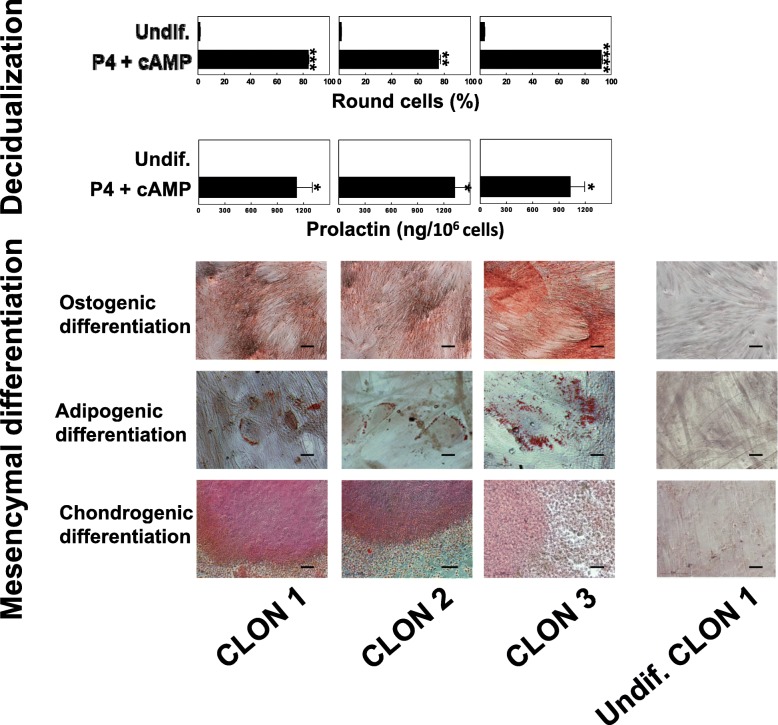
Three preDSC clones exhibited both decidual and mesenchymal characteristics. All three clones were able to decidualize (i.e., to show morphological changes and secrete PRL) and differentiate into osteoblasts, chondrocytes, and adipocytes. Bar graphs show the percentage of round cells and the secretion of PRL by each of the three clones (mean ± SD of three independent determinations per clone). Mesenchymal differentiation was documented with alizarin staining (osteogenic), oil red O solution (adipogenic), and safranin O (chondrogenic). Scale bar, 50 μm. These experiments were done in 4 independent replications. *P < 0.001, **P < 0.005, ***P < 0.0001, ****P < 0.00005. Undifferentiated preDSC clone (Undif.)

Three preDSC clones showed an antigen phenotype equivalent to that expressed by preDSC lines, and also compatible with that of bone marrow MSC [19, 36]. When these three clones were cultured with P4 and cAMP, they also changed their cell morphology toward a rounder shape and secreted PRL. Under appropriate conditions, these three clones also differentiated into adipocytes, chondrocytes, and osteoblasts (Fig. 2). These results demonstrated that the same type of cell can undergo decidual or mesenchymal differentiation.

### PreDSC lines reduced the resorption/implantation ratio in the abortive mating combination female CBA/J × male DBA/2

**Fig. 3:**
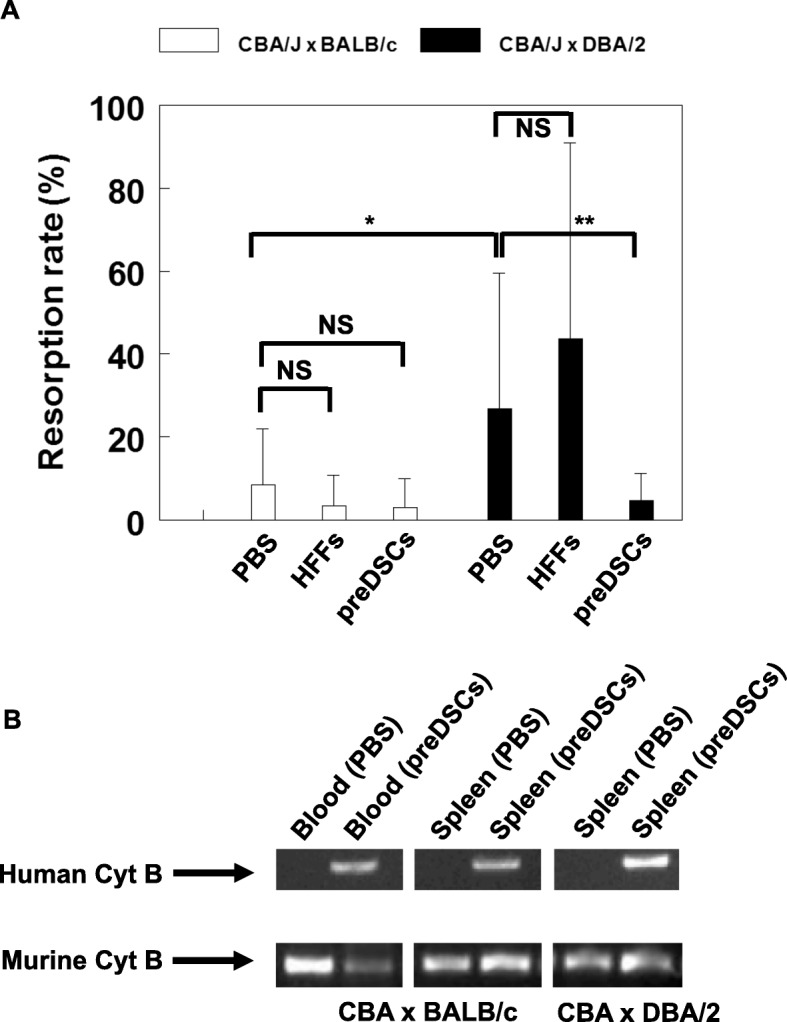
PreDSCs inhibited the resorption rate in the abortion-prone CBA/J♀ × DBA/2♂ combination. preDSCs, HFFs, or PBS were injected on day 2 of gestation in CBA/J mice mated to DBA/2 mice or control BALB/c mice, and the rate of embryo resorption (percentage of reabsorbed embryos referred to the total number of embryo implantations) was determined on day 14 of gestation. **a** The resorption rate (mean ± SD) was significantly higher in the abortion-prone CBA/J♀ × DBA/2♂ combination than in the control CBA/J♀ × BALB/c♂ combination (*P < 0.01). The injection of preDSCs significantly decreased the abortion rate in CBA/J♀ × DBA/2♂ matings (**P < 0.001). The injection of HFFs increased the abortion rate, but not significantly. **b** PreDSCs were detected by human Cyt B PCR in the blood and spleen of BALB/c-mated pregnant CBA/J mice and in the spleen of DBA/2-mated pregnant CBA/J mice in which preDSCs were injected. None of the control mice, in which preDSCs were not injected (PBS), was positive. Murine Cyt B PCR was positive in all samples

To study the in vivo immunoregulatory activity of preDSCs, we injected these cells in the abortion-prone mating combination CBA/J♀ × DBA/2♂. As expected, in this combination, the resorption/implantation ratio was significantly higher than in the nonabortion-prone mating combination CBA/J♀ × BALB/c♂ (*P* < 0.01). The injection of preDSCs in the abortion-prone mating combination led to a significant reduction in the resorption/implantation ratio (*P* < 0.001), whereas control HFFs increased this ratio, although not significantly. The injection of preDSCs and HFFs in the control mating combination had no significant effect (Fig. 3a, Additional file 1: Table S1).

### Detection of human preDSCs injected in immunocompetent mice

The results with RT-PCR for the human marker Cyt B confirmed the presence of preDSCs on day 14 of gestation, i.e., 12 days after the injection of human preDSCs in both control and experimental animals in the murine model of recurrent abortion. These cells were detected in the blood, in the spleen from BALB/c-mated pregnant CBA/J mice, and in the spleen of DBA/2-mated pregnant CBA/J mice (Table 3). Blood and spleen samples from CBA/J mice in which preDSCs were not injected were all negative for human Cyt B, but all samples were positive for murine Cyt B (Fig. 3b). Although human Cyt B might have been detected in murine macrophages that could have phagocytosed preDSCs, it is highly unlikely that preDSC mitochondrial DNA would survive in macrophages for up to 12 days after human preDSCs were injected.

**Table 3:** Detection of preDSCs in tissues of immunocompetent mice in which human preDSCs were injected

Strain	Cells injected/method of detection	Blood	Lymph nodes	Spleen
CBA/J	PreDSCs/RT-PCR	Day 12^a^: 3/3^b^	ND	Day 12: 2/6^c^
BALB/c	PreDSC-GFP/Flow cytometry	Day 2: 3/3 Day 45: 0/3	Day 2: 0/3 Day 45: 1/3	Day 2: 0/3 Day 30: 1/3

*ND* not done

^a^BALB/c-mated pregnant CBA/J

^b^3 positive mice out of 3

^c^1/3 DBA/2-mated pregnant CBA/J and 1/3 in BALB/c-mated pregnant CBA/J

**Fig. 4:**
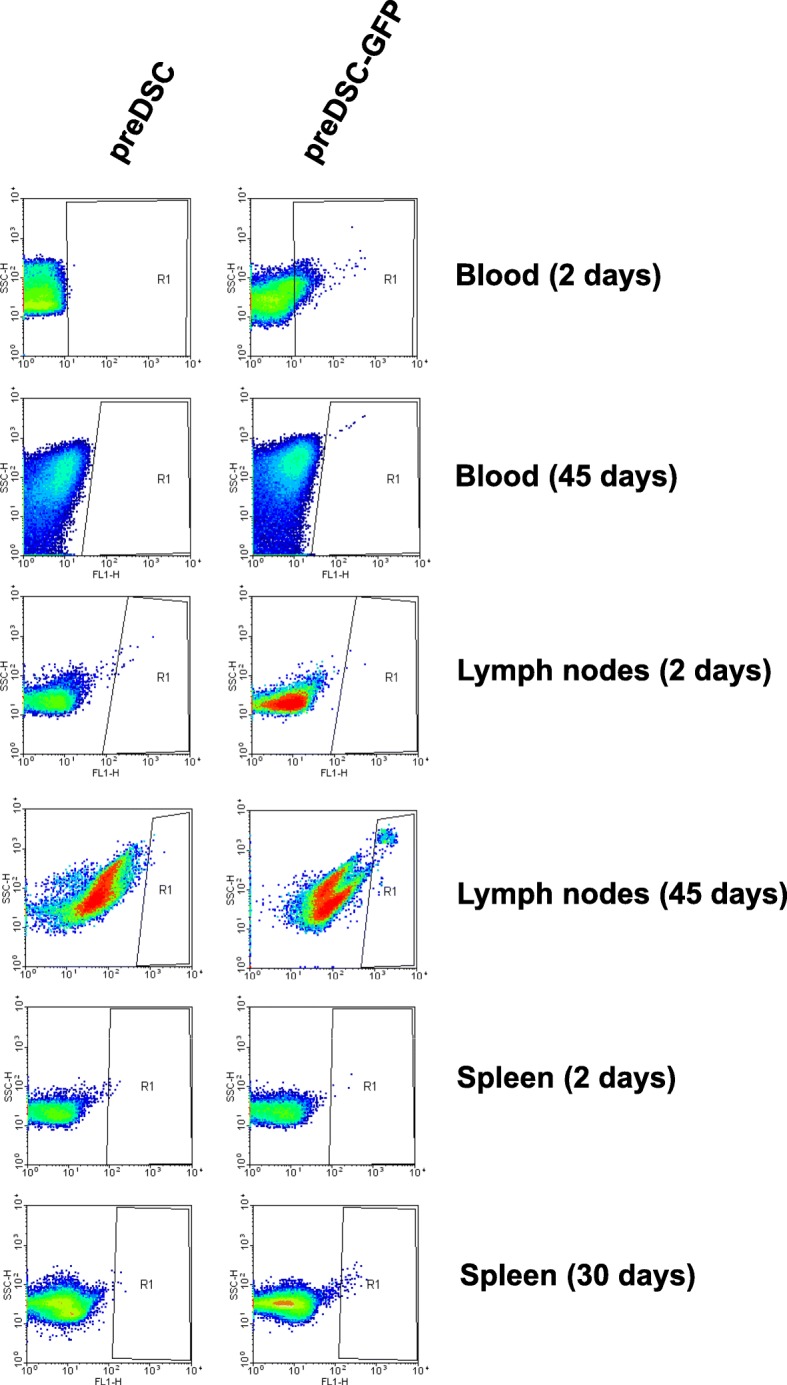
Detection of preDSCs in tissues of mice in which human preDSCs were injected. Human preDSCs transduced with GFP (preDSC-GFP) and injected in BALB/c female mice were detected by flow cytometry in the blood 2 days after the injection and in the inguinal lymph nodes after 45 days. Thirty days after the injection, preDSCs appeared to be present in the spleen although this observation was not conclusive. As a control, we used BALB/c mice in which nontransduced preDSCs were injected

We also confirmed the presence of live preDSCs in virgin female BALB/c mice by injecting GFP-labeled preDSCs. These cells were detected by flow cytometry in the blood after 2 days and in the inguinal lymph nodes after 45 days. Thirty days after the injection, preDSCs appeared to be present in the spleen. PreDSCs were not found in the blood after 45 days, in the inguinal lymph nodes after 2 days, or in the spleen after 2 days (Fig. 4, Table 3). Because the degradation half-life of GFP is only a few hours [44], the detection of this protein in cells days and weeks after the injection in the mice indicates active GFP synthesis by preDSCs that remain alive.

The aim of these experiments was not to establish a precise dynamic of the diffusion of preDSCs in different tissues and organs but to confirm the ability of these cells to survive in the short, middle, and long term in immunocompetent mice. In general, our results showed that preDSCs survived in the blood in the short term (2 days) and in the middle term (12 days) in 100% of mice, but these cells disappeared from this tissue in the long term (45 days). In the lymph nodes and spleen, preDSCs were not detected in the short term; however, these cells were found in the middle (in 2 of out 6 mice) and long term (1 of out 3 mice) in spleen tissue. In all, preDSCs survived in 10 of out 27 mice (Table 3).

## Discussion

Our preDSC lines consisted of a highly purified, uniform, adherent cell population. Under the effects of cAMP and P4, these cells displayed the physiological differentiation pathway (decidualization) of DSCs: their morphology changed from a fibroblastic to a rounder shape, and they began to secrete PRL (Fig. 1a) [8–13]. Furthermore, the antigen phenotype of these preDSC lines was previously studied and found to be fully compatible with that of bone marrow MSCs [5, 19] (Table 1). This antigen phenotype, together with the adherence of preDSCs to plastic culture dishes and their capacity to differentiate into adipocytes, osteoblasts, and chondrocytes (Fig. 1d), are properties that, taken together, meet the minimal criteria proposed by the International Society for Cellular Therapy (ISCT) to define human MSCs [37]. The possibility that these MSC and DSC properties were due to the presence of different cell subpopulations in our preDSC lines was ruled out. The facts that all three of the preDSC clones we obtained exhibited an antigen phenotype like that of preDSC lines [19], and therefore equivalent to that of MSCs, and underwent mesenchymal and decidual differentiation (Fig. 2) demonstrate that single type of cell, i.e., preDSCs, exhibited both DSC and MSC characteristics. Other MSC features are the expression of stem cell markers, clonogenicity, and immunoregulatory activities [32]. Likewise, preDSC lines expressed the multipotentiality markers OCT-4, NANOG, and ABCG2, their clonogenic efficiency ranged between 4 and 15% (Fig. 1), they remained alive for weeks in xenogeneic transplants (Fig. 4), and they had a therapeutic effect in an immune-based murine model of spontaneous abortion (Fig. 3). Taken together, these findings confirm the close relationship between bone marrow MSCs and preDSCs (Table 4).

**Table 4:** Comparison of preDSCs and MSCs

Characteristics	PreDSCs	MSCs	References
Antigen phenotype	Table 1	Table 1	[5, 19, 36]
ISCT minimal criteria	+	+	[19, 37]
Decidualization	PRL (mRNA+, protein+)	PRL (mRNA+, protein−)	[5]
Mesenchymal differentiation	+	+	This work, [37]
Cell contractility	+	+	[19, 29]
Stem cell markers	+	+	This work,[43]
Clonogenicity	+	+	This work, [45]
Apoptosis resistance	+	+	[27, 30]
Hematopoietic cell supportive activity	+	+	[18, 46]
Survival in xenotransplants	+	+	This work, [47, 48]
Perivascular location	+	+	[19, 49]
In vivo and in vitro immunoregulatory activity	+	+	This work [5, 7, 23]

Dimitrov et al., also using human preDSC lines, found an association between these cells and MSCs [31]. This connection suggests that MSCs home to the nongestational endometrium or the decidua to develop into preDSCs. This possibility is supported by the finding of endometrial cells of donor origin in the uterus of women who received bone marrow transplants [50].

A single-cell transcriptomics approach was used by Vento-Tormo et al., who identified different cell types in the first-trimester human placenta. The cellular composition of human decidua includes the presence of perivascular cells, with an antigen phenotype (CD146+ CD140b+, W5C5+, α-SM actin+, CD29+, ANGPT1+, and VCGFA+) consistent with that reported by us for preDSCs, cells that also exhibit a perivascular location (Table 1) [19, 51]. We demonstrated the close relationship between human preDSCs and pericytes [19], the latter also being a perivascular cell type closely associated with—possibly even identical to—MSCs [22–24]. PreDSCs also appear to correspond to the MSCs in the human endometrium reported by other authors (endometrial MSCs, eMSCs), i.e., clonogenic, self-renewing, multipotent cells that can differentiate into adipogenic, osteogenic, chondrogenic, and myogenic lineages [52]. Like preDSCs, eMSCs are CD146+, CD140b+, and W5C5+, decidualize, are found in perivascular sites, and have also been associated with pericytes [19, 53–56]. However, eMSCs were isolated by cell sorting from nongestational endometrium samples, whereas the preDSC lines we obtained from the decidua were enriched by cell culture [19].

Bone marrow or fat MSCs exert immunoregulatory activities that support therapeutic effects against inflammatory and autoimmune diseases [32]. In the present study, we show that preDSCs survived in xenogeneic transplants in vivo for prolonged periods in immunocompetent mice (Fig. 4). This result is consistent with the findings that demonstrated the long-term survival of human MSCs in immunocompetent mice [47] and rats [48]. Although the immunological mechanisms involved in our in vivo experiments were not studied, several lines of experimental evidence confirm that MSCs function across species barriers [57]. In this connection, it has been shown before that human DSCs can interact with the rat and mouse immune systems [58, 59]. The expression of HLA-G and the secretion of IL-10 by preDSCs [3] may be additional mechanisms involved in the induction of tolerance and escape from the immune response in xenotransplants [60, 61]. Other immunoregulatory properties, such as the induction of T regulatory cells (Tregs), detected in MSCs and DSCs [62, 63], and our own unpublished data, may explain the escape from immune rejection in allo- and xenotransplants. In this connection, it was proposed that MSCs play a key role in the physiological mechanisms of immune self-tolerance [23]. Likewise, preDSCs play a role in local mechanisms of maternal–fetal immune tolerance that support normal pregnancy [3, 18]. The abortion-prone mating of female CBA/J mice with male DBA/2 mice is an animal model of human immunologically mediated spontaneous abortion in which the maternal–fetal immune tolerance has broken down [33, 34]. We considered this murine model appropriate to investigate both the potential regulatory effect of preDSCs in immune-based human recurrent abortion and the role of preDSCs in maintaining maternal–fetal immune tolerance. This hypothetical function of preDSCs is supported by our finding that the injection of preDSCs in CBA/J females mated to DBA males significantly decreased the embryo resorption rate (Fig. 3, Additional file 1: Table S1). Similarly, syngeneic murine fat MSCs were shown to be therapeutically effective in this model [64]. Other reports have also documented the beneficial effects of human DSCs in steroid-refractory graft-versus-host disease in humans [65, 66], a finding that identifies DSCs as a potentially important component of cell therapies for immune-mediated diseases. In this connection, the availability of purified, expandable preDSC lines may help to facilitate further research on the clinical applications in the treatment of inflammatory and autoimmune diseases.

## Conclusions

Our data show that preDSCs are decidual MSCs that exhibit immunoregulatory activities that may be relevant in the decidual mechanisms of maternal–fetal immune tolerance. These cells may also be beneficial in the treatment of immune-mediated diseases.

## Additional file

Additional file 1: **Table S1.** Effect of preDSCs on the embryo resorption rate in the abortion-prone CBA/J × DBA/2 combination and control CBA/J × BALB/c combination. (DOC 30 kb)

## Data Availability

All data generated or analyzed during this study are included in this published article.

## Abbreviations

ABCG2: ATP-binding cassette subfamily G member 2
AP: Alkaline phosphatase
B2MG: Beta-2 microglobulin
CE: Cloning efficiency
CMV: Cytomegalovirus
Cyt B: Cytochrome B
DSC: Decidual stromal cells
FCS: Fetal calf serum
GFP: Green fluorescent protein
HFFs: Human foreskin fibroblasts
MSC: Mesenchymal stromal/stem cells
OCT-4: Octamer-binding transcription factor 4
P4: Progesterone
PPARγ2: Peroxisome proliferator-activated receptor γ2
PreDSCs: Precursors of DSC
PRL: Prolactin
RT-PCR: Reverse transcription polymerase chain reaction
VSVg: Vesicular stomatitis virus G glycoprotein
α-SM actin: α-Smooth muscle actin
