# Supplementary material for: Human predecidual stromal cells are mesenchymal stromal/stem cells and have a therapeutic effect in an immune-based mouse model of recurrent spontaneous abortion
Source: Stem Cell Res Ther. 2019 Jun 14;10:177. doi: 10.1186/s13287-019-1284-z (PMC6567662; doi:10.1186/s13287-019-1284-z)
Supplement: Supplementary file 1 — Table S1. Effect of preDSCs on the embryo resorption rate in the abortion-prone CBA/J × DBA/2 combination and control CBA/J × BALB/c combination. (DOC 30 kb) [file 13287_2019_1284_MOESM1_ESM.doc]

**Table S1**. Effect of preDSCs on the embryo resorption rate in the abortion-prone CBA/J x DBA/2 combination and control CBA/J x BALB/c combination.

| Injection |  | CBA/J x DBA/2 | CBA/J x BALB/c |
| --- | --- | --- | --- |
| PBS | Number of mice | 25 | 25 |
|  | R/T^a^ (%) | 52/183 (28.42)* | 15/170 (8.82) |
| preDSCs | Number of mice | 16 | 16 |
|  | R/T (%) | 6/119 (5.04)** | 4/111 (3.60) |
| HFFs | Number of mice | 8 | 9 |
|  | R/T (%) | 28/61 (45.90) | 2/65 (3.08) |

^a^R/T: number of embryo resorptions/total number of embryo implantations. The R/T was significantly higher in the abortion-prone CBA/J x DBA/2 combination than in the control CBA/J x BALB/c combination (**P*<0.01). The injection of preDSCs significantly decreased the abortion rate in CBA/J x DBA/2 (***P*<0.001). The injection of HFFs increased the abortion rate, but not significantly.
